# High PD-L1 expression is associated with therapeutic response to pembrolizumab in patients with advanced biliary tract cancer

**DOI:** 10.1038/s41598-020-69366-4

**Published:** 2020-07-23

**Authors:** Soomin Ahn, Jong-chan Lee, Dong Woo Shin, Jaihwan Kim, Jin-Hyeok Hwang

**Affiliations:** 1 Departments of Pathology, Seoul National University College of Medicine, Seoul National University Bundang Hospital, Seongnam, Gyeonggi Republic of Korea; 2 Department of Internal Medicine, Seoul National University College of Medicine, Seoul National University Bundang Hospital, 82, Gumi-ro 173 Beon-gil, Bundang-gu, Seongnam-si, Gyeonggi-do 13620 Republic of Korea

**Keywords:** Cancer, Diseases, Gastroenterology

## Abstract

Pembrolizumab appears promising for patients with programmed cell death ligand-1 (PD-L1)-positive solid tumors. However, data on immunotherapy for biliary tract cancers (BTC) are limited. We aimed to investigate the predictive value of PD-L1 expression as an immunotherapeutic biomarker in BTC. Patients with advanced BTC (n = 175) were screened for PD-L1 expression using PharmDx assay and microsatellite instability (MSI) status. Of the total of 175 patients, 125 (71%) showed tumoral PD-L1 positivity (≥ 1%) while only two (2/142, 1.4%) showed MSI-High. Among 175 patients, 26 patients were treated with pembrolizumab as a second-line therapy, and tumor response was evaluated. Separating these patients into two groups by PD-L1 expression (high [≥ 50%] vs. low [< 50%]), overall response rate was 23% (56% [5/9] in high PD-L1 group vs. 6% [1/17] in low PD-L1 group, *P* = 0.004). Disease control rate was also higher in high PD-L1 group (78% vs. 35%, *P* = 0.019). The six responders showed median progression-free survival of 5.8 months after starting pembrolizumab, and none of them was MSI-High. High PD-L1 expression was associated with a better response to pembrolizumab. PD-L1 expression can potentially serve as an alternative predictive biomarker for pembrolizumab therapy in advanced BTC.

## Introduction

Biliary tract cancers encompass a heterogeneous group of cancers including those of the intrahepatic and extrahepatic bile duct as well as the gallbladder, and they show dismal clinical outcomes with 5-year survival rates of 20–30%^1^. Most patients are diagnosed at an advanced stage making palliative chemotherapy the only option^1^. Gemcitabine-cisplatin combination is the standard first-line treatment for advanced biliary tract cancer with no standard second-line therapy^2,3^. Several anti-programmed cell death protein 1 (PD-1)/programmed cell death ligand-1 (PD-L1) agents have demonstrated long-lasting anti-tumoral effects in various solid tumors and are now approved for standard treatment in several solid cancers including lung cancer^4,5^. PD-L1 expression has been reported in 9.1–72.2% patients with biliary tract cancer^6–8^; thus, anti-PD-1 or PD-L1 agents can be a promising treatment modality in certain patients with biliary tract cancers. However, immunotherapeutic data on biliary tract cancer are limited compared to other tumors^9,10^. Nevertheless, interim results of an ongoing trial (KEYNOTE-028, NCT02054806) showed promising outcomes of pembrolizumab (anti-PD-1 antibody) in patients with PD-L1-positive advanced biliary tract cancers^9^.

Biomarker development is essential in enhancing efficacy of immunotherapy. For instance, in several tumors including lung and gastric cancers, PD-L1 expression evaluated by immunohistochemistry (IHC) is used as a crucial biomarker predicting response to anti-PD-1/PD-L1 agents^11,12^. PD-L1 IHC (PharmDx assay) has been approved as a companion diagnostic test to select lung cancer patients eligible for pembrolizumab treatment^5^. The approval was based on the observation that patients with high PD-L1 expression showed improved response rates^13^. However, predictive biomarkers remain unknown for the vast majority of other tumors; hence, microsatellite instability (MSI) status, tumor-infiltrating lymphocytes (TIL), tumor mutational burden, and several other factors are being investigated as candidate immunotherapeutic biomarkers^14^. Pembrolizumab was recently approved by the Food and Drug Administration for treatment of patients with metastatic or unresectable mismatch repair (MMR)-deficient and/or MSI-High (MSI-H) solid tumors that progressed after prior therapy regardless of tumor type^15^. However, MSI-H biliary tract cancers are rare^16^. To date, studies on immunotherapeutic biomarkers in biliary tract cancers are limited. In this study, we evaluated therapeutic response of advanced biliary tract cancer patients to pembrolizumab, and correlated treatment response with biomarkers including PD-L1 IHC.

## Results

### PD-L1 and MSI screening

**Figure 1:**
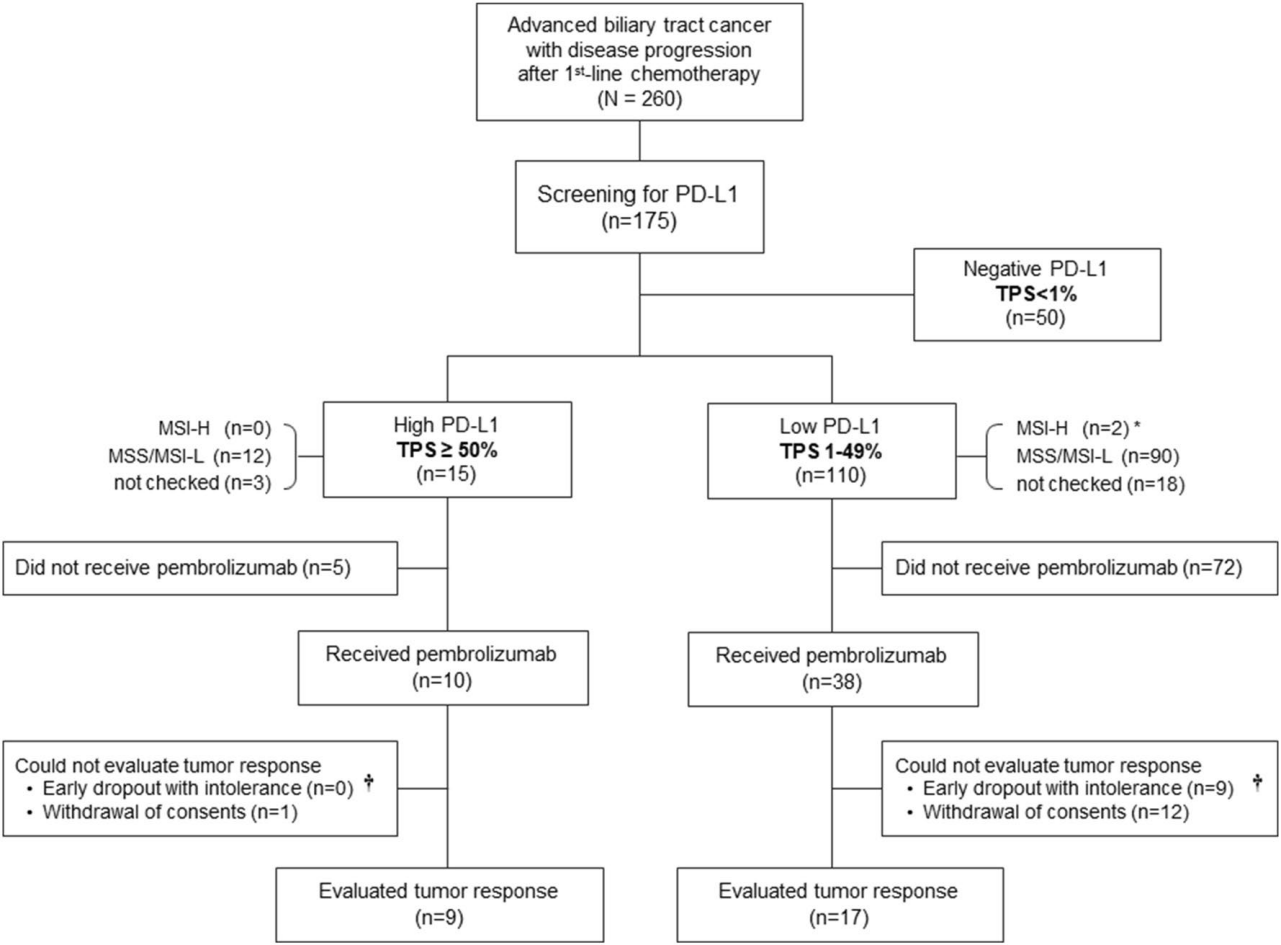
Patient flowchart. *Among the two MSH-H patients, only one received pembrolizumab treatment and showed disease progression. ^†^These patients were only included in ‘toxicity analysis’, and not in ‘efficacy analysis.’

In the 175 patients, the primary tumor site was the intrahepatic bile duct in 55 (31%) patients, hilar bile duct in 39 (22%), distal extrahepatic bile duct in 18 (10%), gallbladder in 56 (32%), and ampulla of Vater in 7 (4%) patients. Among them, 71.4% (125 of 175) of patients showed tumoral PD-L1 positivity at 1% cut off. High PD-L1 expression (tumor proportion score [TPS] ≧ 50) was present in 8.6% (15 of 175) of patients. MSI status was evaluated in 142 patients, and only 2 (1.4%) out of 142 had MSI-H status as revealed by polymerase chain reaction (PCR) assay. These two MSI-H tumors showed low PD-L1 expression (TPS = 1 and 5). One patient received pembrolizumab and the other did not. The one who received pembrolizumab showed progressive disease. The PD-L1 IHC and MSI status are summarized in Table 1. Overall patient flowchart is shown in Fig. 1.

**Table 1:** PD-L1 and MSI status in screened 175 biliary tract cancer patients.

	PD-L1 level (%)			MSI status		
	Negative (TPS = 0)	Low (TPS 1–49)	High (TPS ≥ 50)	Not done	MSS or MSI-low	MSI-high
Intrahepatic, n = 55 (31%)	12 (7%)	38 (22%)	5 (3%)	8 (5%)	47 (27%)	0 (0%)
Hilar, n = 39 (22%)	17 (10%)	21 (12%)	1 (1%)	11 (6%)	26 (15%)	2 (1%)*
Extrahepatic, n = 18 (10%)^†^	2 (1%)	15 (9%)	1 (1%)	2 (1%)	16 (9%)	0 (0%)
Gallbladder, n = 56 (32%)	16 (9%)	32 (18%)	8 (5%)	11 (6%)	45 (26%)	0 (0%)
AoV, n = 7 (4%)	3 (2%)	4 (2%)	0 (0%)	1 (1%)	6 (3%)	0 (0%)
Overall, n = 175 (100%)	50 (29%)	110 (63%)	15 (9%)	33 (19%)	140 (80%)	2 (1%)

PD-L1, programmed cell death ligand 1; TPS, tumor proportion score; MSI, microsatellite instability; MSS, microsatellite stable; AoV, Ampulla of Vater.

*These two patients showed low PD-L1 level.

^†^Extrahepatic bile duct cancer excluding hilar bile duct cancer.

### Treatment outcome

Between Feb 2018 and June 2019, 26 patients among PD-L1 positive patients (≥ 1%) were treated with pembrolizumab as a second-line therapy and they were evaluated the tumor response by computed tomography (CT) or magnetic resonance imaging (MRI). The median age was 67 years with an age range of 41 to 86 years. The ratio of sex was equal, and all were of Asian ethnicity. All patients had received prior standard chemotherapy and had been found refractory. The median number of cycles was 4 as of Dec 2019. The median follow-up duration was 13.1 months. Patient information is summarized in Table 2.

**Table 2:** Characteristics at the time of starting pembrolizumab.

	High PD-L1 (n = 9)	Low PD-L1 (n = 17)	Total (n = 26)	*P*-value
Age, median (range)	70 (59–74)	61 (41–86)	67 (41–86)	0.372
Female	5 (56%)	8 (47%)	13 (50%)	0.680
Location of tumor*				0.427
Intrahepatic	3 (33%)	7 (41%)	10 (38%)	
Hilar (Klatskin)	0 (0%)	3 (18%)	3 (12%)	
Extrahepatic^†^	2 (22%)	3 (18%)	5 (19%)	
Gallbladder	4 (44%)	3 (18%)	7 (27%)	
AoV	0 (0%)	1 (6%)	1 (4%)	
Stage				0.960
Localized	1 (11%)	2 (12%)	3 (12%)	
Metastatic	8 (89%)	15 (88%)	23 (88%)	
Number of metastatic organ				0.272
0	1 (11%)	2 (12%)	3 (12%)	
1	0 (0%)	5 (29%)	5 (19%)	
2	6 (67%)	6 (35%)	12 (46%)	
≥ 3	2 (22%)	4 (24%)	6 (23%)	
ECOG performance score				0.648
0–1	5 (56%)	11 (65%)	16 (62%)	
≥ 2	4 (44%)	6 (35%)	10 (38%)	
Tumor marker, median (IQR)				
CEA	5.0 (1.8–13.2)	3.5 (1.9–19.9)	5.0 (1.9–23.7)	0.572
CA 19–9	141 (29–1900)	260 (50–950)	220 (29–1,360)	0.335
Level of PD-L1				
median % (range %)	80 (60–95)	2 (1–30)	5 (1–95)	< 0.001
MSI status				0.548
MSI-high	0 (0%)	1 (6%)	1 (4%)	
MSS or MSI-low	8 (89%)^§^	16 (94%)	24 (92%)^§^	

PD-L1, programmed cell death ligand 1; AoV, Ampulla of Vater; ECOG, Eastern Cooperative Oncology Group; IQR, interquartile range; MSI, microsatellite instability; MSS, microsatellite stable.

*Epicenter of the tumor, ^†^Extrahepatic bile duct cancer excluding hilar bile duct cancer, ^§^The result of one patient was not available.

**Figure 2:**
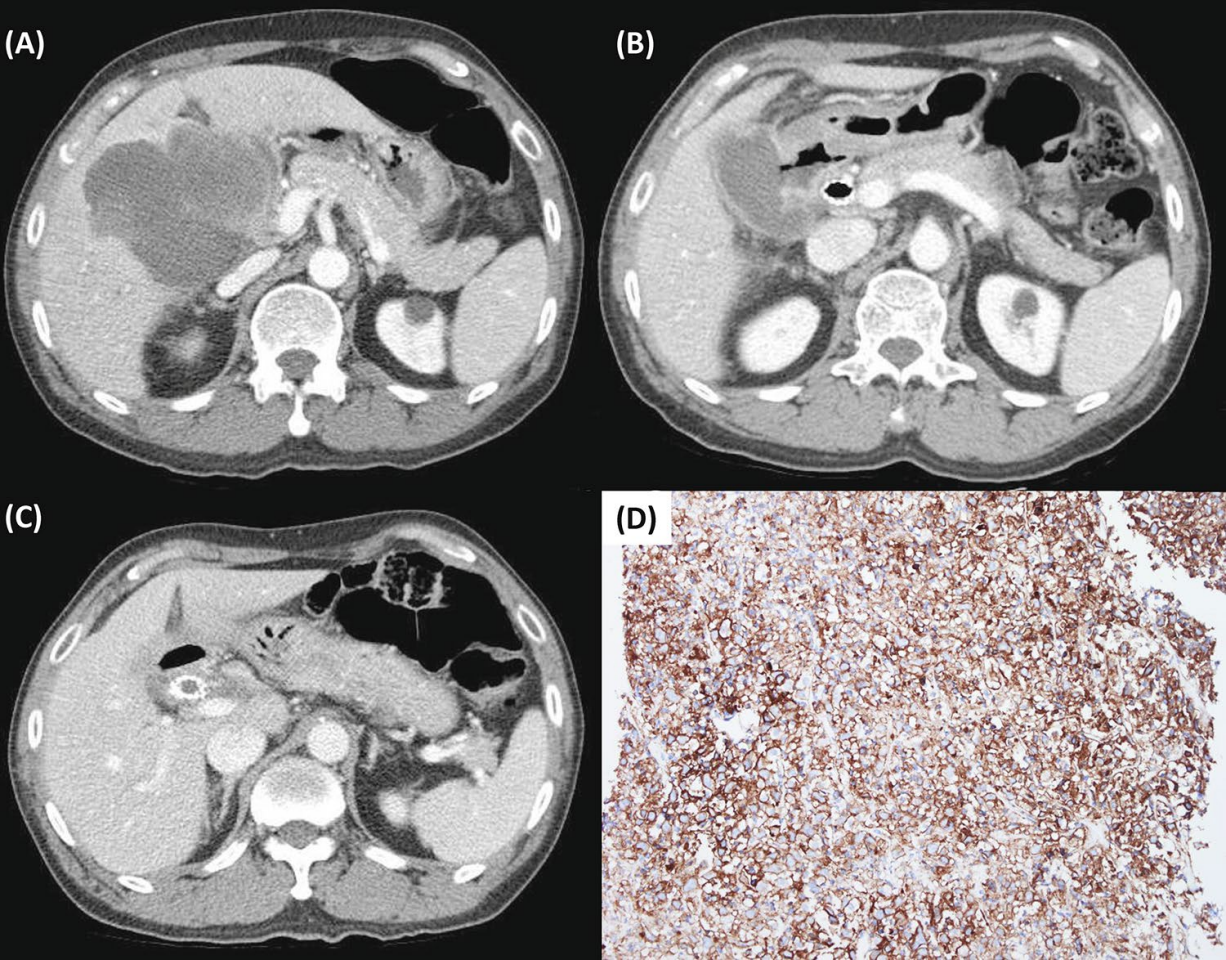
Representative images of abdominopelvic computed tomography and immunohistochemistry from one responder with gallbladder cancer (TPS = 80). (**A**) Baseline computed tomography scan. (**B**) Computed tomography scan after 3 cycles of pembrolizumab. (**C**) Computed tomography scan after 10 cycles of pembrolizumab. (**D**) PD-L1 immunohistochemistry showing high PD-L1 expression (20 × magnification).

As listed in Table 3, no patient achieved complete remission. A total of six (23%) patients showed partial response. Seven (27%) patients showed stable disease. The overall tumor response rate (ORR) was 23% and was statistically different between the two groups (56% [5/9] in high PD-L1 group vs. 6% [1/17] in low PD-L1 group, *P* = 0.004). Also, disease control rate (DCR) was 50% and showed a statistically significant difference between the two groups (78% [7/9] high PD-L1 group vs. 35% [6/17] low PD-L1 group, *P* = 0.019). The computed tomography and IHC images of one of the responders belonging to high PD-L1 group are shown in Fig. 2.

**Table 3:** Treatment outcomes of pembrolizumab.

	High PD-L1 (n = 9)	Low PD-L1 (n = 17)	Total (n = 26)	*P*-value
Cycles				0.016
Median (range)	7 (3–20)	5 (3–9)	6 (3–20)	
Best response				
CR	0 (0%)	0 (0%)	0 (0%)	
PR	5 (56%)	1 (6%)	6 (23%)	
SD	2 (22%)	5 (29%)	7 (27%)	
PD	2 (22%)	11 (65%)	13 (50%)	
Tumor response rate				
Overall response (ORR, %)*	5 (56%)	1 (6%)	6 (23%)	0.004
Disease control (DCR, %)^†^	7 (78%)	6 (35%)	13 (50%)	0.019

PD-L1, programmed cell death ligand 1; CR, complete response; PR, partial response; SD, stable disease; PD, progressive disease; ORR, overall response rate; DCR, diseased control rate.

*ORR = (CR + PR)/(CR + PR + SD + PD)*100 (%).

^†^DCR = (CR + PR + SD)/(CR + PR + SD + PD)*100 (%).

The six responders including five in high PD-L1 group and one in low PD-L1 group showed median progression-free survival of 5.8 months (range 1.8–14.5 months) after starting pembrolizumab, and none of them were MSI-H.

Additional analyses were performed for PD-L1 positive patients who did not receive pembrolizumab. First, in all of the patients with high PD-L1, patients treated with pembrolizumab showed a significant improvement in survival (HR 0.423, 95% CI 0.249–0.952). However, initial patient status such as Eastern Cooperative Oncology Group performance and carbohydrate antigen 19–9 showed different trends in the treated and untreated patients (Supplementary Table 1). Second, in all of the patients without treatment, the high (n = 5) vs low (n = 72) PD-L1 group did not show a significant difference in clinical characteristics (Supplement Table 2).

### Toxicity profile

The patients who were evaluated for tumor response (n = 26) and who dropped out early because of toxicity or intolerance (n = 9) were assessed for toxicity profile (Fig. 1 and Table 4). Generally, overall treatment-related adverse events including (1) hematologic, (2) other laboratory, and (3) clinical adverse effects did not show a significant difference between the two groups. Unlike other cytotoxic chemotherapeutic agents, neutropenia was relatively low (grade I-II 6%, grade III-IV 3%). Grade I-II hypercalcemia was present in five (14%) patients. Overall, alanine transferase (ALT) elevation was observed in nine (25%) patients: grade I-II 14% and grade III-IV 11%.

**Table 4:** Treatment-related adverse events.

	Grade I–II (n = 35)	Grade III–IV (n = 35)
Neutropenia	2 (6%)	1 (3%)
Anemia	3 (9%)	2 (6%)
ALT elevation	5 (14%)	4 (11%)
Hypercalcemia	5 (14%)	0 (0%)
Nausea/vomiting	4 (11%)	4 (11%)
Fatigue	7 (20%)	4 (11%)
Decreased appetite	3 (9%)	1 (3%)
Constipation	3 (9%)	2 (6%)
Diarrhea	2 (6%)	2 (6%)
Rash	1 (3%)	0 (0%)
Pruritus	7 (20%)	0 (0%)
Pyrexia	7 (20%)	1 (3%)
Peripheral edema	4 (11%)	0 (0%)
Others	0 (0%)	0 (0%)

ALT, alanine transferase.

*Others include thrombocytopenia, mucositis, arthritis, balance disorder, cough, ascites, deep vein thrombosis, pulmonary thromboembolism, ophthalmological problems.

### Association of response with biomarkers

As described above, high-PD-L1 group showed ORR of 56% while low PD-L1 group showed ORR of 6% (*P* = 0.004). DCR was 78% vs. 35% (high PD-L1 group vs. low PD-L1 group, *P* = 0.019). PD-L1 TPS and combined positive score (CPS) showed the same results as shown in Supplementary Table 3. Only one patient with MSI-H status (in low PD-L1 group) was treated with pembrolizumab and showed progressive disease. Using TIL level as a biomarker, high TIL level (≥ 7.5%, median value) and low TIL level (< 7.5%) showed 36% vs. 13% in ORR (*P* = 0.251) and 55% vs. 47% in DCR (*P* = 0.922). Using CD^8+^ cells level as a biomarker, high CD^8+^ cells count (≥ 50, median value) and low CD^8+^ cells count (< 50) showed 36% vs. 17% in ORR (*P* = 0.355) and 55% vs. 58% in DCR (*P* = 0.834).

## Discussion

Immunotherapy has emerged as a new paradigm in cancer treatment. Pembrolizumab, a monoclonal antibody against PD-1, has shown long-lasting antitumor activity and manageable toxicity in several advanced cancers^4^. An ongoing phase 1b clinical trial showed promising clinical results with pembrolizumab treatment in advanced PD-L1-positive biliary tract cancers^9^. In the trial, PD-L1 positivity was defined as staining in 1% of cells in tumor nests or PD-L1-positive bands in the stroma by the 22C3 assay, and the ORR was 17.4%^9^.

In our study, 6 (23%) of the 26 patients with PD-L1-positive (TPS ≥ 1%) biliary tract cancers revealed partial response to pembrolizumab, and 7 (27%) showed stable disease. Notably, the responders were mostly enriched in high PD-L1 expression although they did not have MSI-H tumors. Evaluating treatment responses to pembrolizumab is clinically significant since currently, there is no effective second-line treatment agent for patients with biliary tract cancer refractory to standard chemotherapy. High PD-L1 expression was significantly associated with response to pembrolizumab in patients with biliary tract cancer, a finding in line with previous studies on other tumors such as lung cancer^11^. To the best of our knowledge, this is the first study reporting correlation between PD-L1 expression level and response to pembrolizumab in biliary tract cancers. Given the dramatic therapeutic response of the patients with high PD-L1 expression in our study, and the fact PD-L1 IHC is considered a cost-effective screening tool for routine usage in the clinic, PD-L1 could serve as an alternative predictive biomarker for pembrolizumab immunotherapy in advanced biliary tract cancers refractory to standard chemotherapy.

Regarding treatment-related adverse effects, pembrolizumab was generally well-tolerated as reported in a previous study^9^. The most-common grade III-IV adverse effects were elevated hepatic enzyme (ALT) level and fatigue. However, most of the adverse effects were manageable and reversible; thus, employing pembrolizumab in biliary tract cancer patients seems feasible.

Recently, pembrolizumab has been approved for MSI-H/MMR-deficient solid tumors regardless of tumor type^15^. In a recent trial of pembrolizumab in patients with previously treated, advanced noncolorectal MSI-H/MMR-deficient tumors encompassing 27 different solid tumors (KEYNOTE-158), 22 patients with biliary tract cancer were included, and the ORR of those biliary tract cancer patients was 40.9%^17^. However, the frequency of MSI-H in biliary tract cancer, which is generally very low^1^, varied from 1 to 10% in different reports^18–20^. In the present study, MSI-H was only observed in 2 (1.4%) of the 142 patients. Given the rarity of MSI-H in biliary tract cancer, we recommend PD-L1 expression to be evaluated along with MSI status when considering immunotherapy. We also correlated other biomarkers including CD^8+^ cell density and TILs with treatment response and found no significant associations. Among various biomarkers, only PD-L1 expression level showed reliable predictability with statistical significance. This supports the argument that PD-L1 IHC can be a useful biomarker for pembrolizumab treatment.

This study has several limitations. First, the sample size was relatively small to draw a definitive conclusion on the usefulness of the biomarkers. In addition, patients with PD-L1 negative biliary tract cancers were not included in this study. Thus, large-scale studies are warranted to determine the role of PD-L1 as a predictive marker of response to pembrolizumab in biliary tract cancers. Second, the enrollment criteria were not strict. Patients with advanced biliary tract cancer are relatively common in South Korea compared to western countries, and the Korean Ministry of Food and Drug Safety approved the usage of pembrolizumab based on the interim result of the ongoing phase 1b clinical trial^9^. Third, the follow-up period was insufficient to evaluate durable treatment effects of pembrolizumab, long-term disease-free survival, and overall survival. However, the responders in high PD-L1 group showed comparable progression-free survival compared to previous studies^17^. Fourth, MSI status was not evaluated in all patients. MSI status was evaluated in 142 out of total 175 patients. In addition, the number of MSI-H tumors was too small to determine the association between MSI status and response to pembrolizumab in biliary tract cancers. However, the very low frequency of MSI-H patients suggests less usefulness of MSI in the clinical setting, which is precisely the reason that novel biomarkers are needed. Lastly, tumor mutation burden, which is a promising biomarker for predicting response to immunotherapy^21^, was not evaluated in this study.

In conclusion, 8.6% of advanced biliary tract cancer patients revealed high PD-L1 expression, and MSI-H tumors were rare (1.4%) in our screening cohort. High PD-L1 expression was associated with treatment response to pembrolizumab. Although further prospective studies using a large cohort is required, PD-L1 expression can potentially serve as a valuable biomarker in identifying those patients with advanced biliary tract cancer who could benefit from pembrolizumab treatment**.**

## Methods

### Patients

Advanced biliary tract cancer patients who experienced disease progression after 1st-line chemotherapy (n = 260) were retrospectively reviewed. Among them, a total of 175 patients were screened for PD-L1 expression and MSI status at Seoul National University Bundang Hospital (Seongnam, Korea) from Feb 2018 to June 2019. All patients had a histologically confirmed diagnosis of either the gallbladder, bile duct, or ampulla of Vater cancer. Pembrolizumab was then administered solely to patients with PD-L1 positive (≥ 1%) tumors that were refractory to standard chemotherapy (gemcitabine with cisplatin), and those who could afford the high cost of treatment (The Ministry of Food and Drug Safety in South Korea approved the usage of pembrolizumab as a second-line chemotherapy in biliary tract cancers patients with positivity of PD-L1 without reimbursement since December 2017).

### Study design

We divided patients treated with pembrolizumab into two groups (high PD-L1 group: PD-L1 expression ≧ 50% vs. low PD-L1 group: PD-L1 expression < 50%) and compared clinical outcomes in these two groups. The primary outcome of this study encompassed ORR and DCR with respect to PD-L1 expression level.

Pembrolizumab was administered intravenously at a fixed dose of 200 mg every 3 weeks up to the point of disease progression which was evaluated following the RECIST criteria version 1.1^22^. Response evaluation was made by investigator assessment. To evaluate the primary outcome (ORR and DCR), the patients who could be evaluated for tumor response (via CT or MRI) were included the ‘efficacy analysis’. Patients who dropped out before evaluating tumor response were excluded in the ‘efficacy analysis’. However, among this excluded group, those who dropped out due to medical reasons such as toxicity or intolerance were included in the ‘toxicity analysis’. Patients who withdrew consent for the reasons other than medical reasons, such as financial problems or access to hospitals, were excluded in both ‘efficacy analysis’ and ‘toxicity analysis’.

The study was approved by the institutional review board of Seoul National University Bundang Hospital (Protocol No.B-1901/519-104), and informed consent was waived. All procedures performed in this study were in accordance with the ethical standards of the institutional research committee and with the 1964 Helsinki declaration and its later amendments or comparable ethical standards.

### PD-L1 immunohistochemistry

Formalin-fixed, paraffin-embedded tissue sections of biopsy or surgical specimens were used for IHC. The PharmDx assay (Dako, Carpinteria, CA, USA) was utilized, using an anti-PD-L1 22C3 mouse monoclonal primary antibody and EnVision FLEX visualization system (Agilent, Santa Clara, CA, USA) on an Autostainer Link 48 system (Dako, Carpinteria, CA, USA) along with negative control reagents and cell line run controls, as per the manufacturers’ instructions^23,24^. PD-L1 staining was defined as complete or partial circumferential linear cellular membrane staining at any intensity that could be differentiated from the background as well as diffuse cytoplasmic staining^23,25^. Tonsillar tissue was used as a positive internal control^23^. Positivity of PD-L1 status was determined based on a 1% threshold in tumor cells. A TPS was recorded as the overall percentage of PD-L1 stained tumor cells relative to the entire tumor area^26^. A CPS was recorded based on the number of PD-L1 positive tumor and immune cells in relation to total tumor cells^26^.

### Microsatellite instability status

The patients’ MSI status was assessed by comparing the allele profiles of 5 tumor cell markers (BAT-26, BAT-25, D5S346, D17S250, and S2S123) to those in the available corresponding normal samples. Hematoxylin and eosin-stained slides were reviewed to select appropriate areas with sufficient tumor cellularity and adequate non-neoplastic tissue for macro-dissection (where applicable), and subsequently, DNA extraction was performed. PCR amplification of the extracted DNA was carried out and the PCR products were analyzed using a DNA autosequencer (ABI 3731 Genetic Analyzer; Applied Biosystems, Foster City, CA, USA). According to the Revised Bethesda Guidelines, tumors with additional alleles in ≥ 2 markers were classified as MSI-H, those with novel bands in 1 marker were defined as MSI-low, and those with identical bands in all 5 markers were classified as microsatellite stable^27^.

### Quantification of tumor infiltrating lymphocytes (TILs) and CD8^+^ lymphocytes

TILs and CD^8+^ lymphocyte analysis was performed by one experienced pathologist (S.A). TILs were scored as a percentage of immune cells in tumor-associated stromal areas^28^. All mononuclear cells (including lymphocytes and plasma cells) were scored; polymorphonuclear leukocytes were excluded^28^. CD^8+^ stained slides were scanned using a high-resolution digital slide scanner up to 400 × magnification (3DHISTECH Pannoramic 250; 3DHISTECH Ltd., Budapest, Hungary), and an area of 1 mm^2^ was marked per tumor. For tumors with heterogeneous distribution of CD^8+^ lymphocytes, one hotspot area was selected per sample. The absolute number of CD^8+^ cells was manually counted.

### CD8 and mismatch repair protein immunohistochemistry

Immunohistochemical staining was performed using the BenchMark XT autostainer (Ventana Medical Systems, Tucson, AZ, USA) with an UltraView detection kit (Ventana Medical Systems, Basel, Switzerland). Commercially-available antibodies were used for immunohistochemical staining; CD8 (Clone C8/144B, ready to use; Dako, Carpinteria, CA, USA), hMLH1 (Clone M1, ready to use; Ventana, Basel, Switzerland), hMLH2 (Clone G219-1,129, 1:100 dilution; Abnova, Taipei City, Taiwan), hMLH6 (Clone 44, 1:100 dilution; Cell Marque, Rocklin, CA, USA), and PMS2 (Clone A16-4, ready to use; Ventana Medical Systems, Basel, Switzerland). The normal lymph node was used as positive control of CD8 stain. For MLH1, MLH2, MLH6, and PMS2, inflammatory cells and stromal tissue were used as internal positive control.

### Statistical analyses

All continuous variables were analyzed using t-test or Mann–Whitney U test depending on normal distribution of the dataset. The range of continuous variables was expressed using whole ranges (minimal to maximal value) with several exceptions. Dichotomous or categorical variables were compared using Chi-square test or Fisher’s exact test. Statistical analyses were performed using STATA version 15.0 (StataCorp, College Station, TX, USA) and SPSS version 25.0 (SPSS, Inc., Chicago, IL, USA). *P*-values < 0.05 were considered significant.

## Supplementary information

Supplementary information.
