# Author Correction: High PD-L1 expression is associated with therapeutic response to pembrolizumab in patients with advanced biliary tract cancer

**DOI:** 10.1038/s41598-020-78512-x

**Published:** 2020-12-03

**Authors:** Soomin Ahn, Jong- chan Lee, Dong Woo Shin, Jaihwan Kim, Jin‑ Hyeok Hwang

**Affiliations:** 1 Departments of Pathology, Seoul National University College of Medicine, Seoul National University Bundang Hospital, Seongnam, Gyeonggi Republic of Korea; 2 Department of Internal Medicine, Seoul National University College of Medicine, Seoul National University Bundang Hospital, 82, Gumi-ro 173 Beon-gil, Bundang-gu, Seongnam-si, Gyeonggi-do 13620 Republic of Korea

Correction to: *Scientific Reports* 10.1038/s41598-020-69366-4, published online 23 July 2020

The article contains an error in Reference 14, Reference 14 is using the incorrect DOI URL. The correct one

should read:

https://doi.org/10.1200/EDBK_160766

