# Supplementary material for: High PD-L1 expression is associated with therapeutic response to pembrolizumab in patients with advanced biliary tract cancer
Source: Sci Rep. 2020 Jul 23;10:12348. doi: 10.1038/s41598-020-69366-4 (PMC7378166; doi:10.1038/s41598-020-69366-4)
Supplement: Supplementary file 1 — Supplementary information. [file 41598_2020_69366_MOESM1_ESM.docx]

**High PD-L1 expression is associated with therapeutic response to pembrolizumab in patients with advanced biliary tract cancer**

Soomin Ahn^1*^, Jong-chan Lee^2*,^ Dong Woo Shin^2^, Jaihwan Kim^2^, Jin-Hyeok Hwang^2^

^1^Departments of Pathology, ^2^Departments of Internal Medicine, Seoul National University College of Medicine, Seoul National University Bundang Hospital, Seongnam, Gyeonggi, Republic of Korea.

**Running title: Pembrolizumab in biliary tract cancers**

**Correspondence:** Jin-Hyeok Hwang, MD, PhD

Department of Internal Medicine, Seoul National University College of Medicine, Seoul National University Bundang Hospital, 82, Gumi-ro 173 Beon-gil, Bundang-gu, Seongnam-si, Gyeonggi-do 13620, Republic of Korea.

Tel.: +82-31-787-7017

Fax: +82-31-787-4051

E-mail: [woltoong@snu.ac.kr](mailto:woltoong@snu.ac.kr).

*S.A. and J-c.L. contributed equally to this work

**Supplementary Table 1. All of the patients who showed high PD-L1 level**

|  | **Untreated**  **(n=5)** | **Treated**  **(n=9)** | **Total**  **(n=14)** | ***P-value*** |
| --- | --- | --- | --- | --- |
| Age, median (range) | 71 (67–80) | 70 (59–74) | 70 (58–83) | *0.366* |
| Female | 1 (20%) | 5 (56%) | 6 (42%) | *0.077* |
| Location of tumor* |  |  |  | *0.270* |
| Intrahepatic | 2 (40%) | 3 (33%) | 5 (36%) |  |
| Hilar (Klatskin) | 1 (20%) | 0 (0%) | 1 (7%) |  |
| Extrahepatic ^†^ | 0 (0%) | 1 (11%) | 1 (7%) |  |
| Gallbladder | 2 (40%) | 5 (56%) | 1 (7%) |  |
| AoV | 0 (0%) | 0 (0%) | 0 (3%) |  |
| Stage |  |  |  | *0.441* |
| Localized | 0 (11%) | 1 (11%) | 1 (7%) |  |
| Metastatic | 5 (100%) | 8 (89%) | 13 (93%) |  |
| Number of metastatic organ |  |  |  | *0.242* |
| 0 | 0 (11%) | 1 (11%) | 1 (8%) |  |
| 1 | 1 (0%) | 0 (0%) | 1 (8%) |  |
| 2 | 2 (40%) | 6 (67%) | 8 (57%) |  |
| ≥ 3 | 2 (40%) | 2 (22%) | 4 (29%) |  |
| ECOG performance score |  |  |  | *0.301* |
| 0–1 | 1 (20%) | 5 (56%) | 6 (42%) |  |
| ≥ 2 | 4 (80%) | 4 (44%) | 8 (57%) |  |
| Tumor marker, median (IQR) |  |  |  |  |
| CEA | 6.2 (2.5–10.2) | 5.0 (1.8–13.2) | 5.1 (3.5–15.7) | *0.631* |
| CA 19-9 | 312 (40–566) | 141 (29–1900) | 250 (56–1210) | *0.092* |
| Overall survival |  |  |  | *0.023* |
| median (range, month) | 2.3 (1.9–8.5) | 9.4 (2.2–18.4) | 5.7 (1.9–18.4) |  |
| hazard ratio (95% CI) | – | 0.423  (0.249–0.952) |  |  |

* Epicenter of the tumor, †Extrahepatic bile duct cancer excluding hilar bile duct cancer,

PD-L1, programmed cell death ligand 1; AoV, Ampulla of Vater; ECOG, Eastern Cooperative Oncology Group; IQR, interquartile range; CI, confidence interval

**Supplementary Table 2. PD-L1 positive patients who did not receive pembrolizumab**

|  | **High PD-L1**  **(n=5)** | **Low PD-L1**  **(n=72)** | **Total**  **(n=77)** | ***P-value*** |
| --- | --- | --- | --- | --- |
| Age, median (range) | 71 (67–80) | 69 (55–86) | 69 (58–85) | *0.494* |
| Female | 1 (20%) | 33 (46%) | 40 (52%) | *0.261* |
| Location of tumor* |  |  |  | *0.670* |
| Intrahepatic | 2 (40%) | 23 (32%) | 25 (32%) |  |
| Hilar (Klatskin) | 1 (20%) | 14 (19%) | 15 (19%) |  |
| Extrahepatic ^†^ | 0 (0%) | 10 (14%) | 10 (13%) |  |
| Gallbladder | 2 (40%) | 23 (32%) | 25 (32%) |  |
| AoV | 0 (0%) | 2 (3%) | 2 (3%) |  |
| Stage |  |  |  | *0.471* |
| Localized | 0 (11%) | 6 (8%) | 6 (8%) |  |
| Metastatic | 5 (100%) | 66 (92%) | 71 (92%) |  |
| Number of metastatic organ |  |  |  | *0.938* |
| 0 | 0 (11%) | 6 (8%) | 6 (8%) |  |
| 1 | 1 (0%) | 14 (19%) | 5 (6%) |  |
| 2 | 2 (40%) | 35 (47%) | 12 (16%) |  |
| ≥ 3 | 2 (40%) | 17 (24%) | 6 (8%) |  |
| ECOG performance score |  |  |  | *0.648* |
| 0–1 | 1 (20%) | 17 (24%) | 18 (23%) |  |
| ≥ 2 | 4 (80%) | 55 (76%) | 59 (77%) |  |
| Tumor marker, median (IQR) |  |  |  |  |
| CEA | 6.2 (2.5–10.2) | 7.5 (3.5–24.1) | 5.0 (3.4–23.7) | *0.416* |
| CA 19-9 | 312 (40–566) | 451 (55–829) | 430 (55–829) | *0.285* |
| Overall survival |  |  |  | *0.491* |
| median (range, month) | 2.3 (1.9–8.5) | 2.8 (0.1–16.7) | 2.7 (0.1–16.7) |  |
| hazard ratio (95% CI) | – | 1.162  (0.529–1.447) |  |  |

* Epicenter of the tumor, †Extrahepatic bile duct cancer excluding hilar bile duct cancer,

PD-L1, programmed cell death ligand 1; AoV, Ampulla of Vater; ECOG, Eastern Cooperative Oncology Group; IQR, interquartile range; CI, confidence interval

**Supplementary Table 3. Best Responses by Various Biomarkers**

|  | **PD-L1**  **TPS** | **(n=26)** |  | **PD-L1**  **CPS** | **(n=26)** |  | **TIL** | **(n=26)** |  | **CD8+cell** | **(n=23)** |
| --- | --- | --- | --- | --- | --- | --- | --- | --- | --- | --- | --- |
|  | **High**  **TPS≥50**  **(n=9)** | **Low**  **TPS<50**  **(n=17)** |  | **High**  **CPS≥70**  **(n=9)** | **Low**  **CPS<70**  **(n=17)** |  | **High**  **TIL≥7.5**  **(n=11)** | **Low**  **TIL<7.5**  **(n=15)** |  | **High**  **CD8≥50**  **(n=11)** | **Low**  **CD8<50**  **(n=12)** |
| Best response |  |  |  |  |  |  |  |  |  |  |  |
| CR | 0 (0%) | 0 (0%) |  | 0 (0%) | 0 (0%) |  | 0 (0%) | 0 (0%) |  | 0 (0%) | 0 (0%) |
| PR | 5 (56%) | 1 (6%) |  | 5 (56%) | 1 (6%) |  | 4 (36%) | 2 (13%) |  | 4 (36%) | 2 (17%) |
| SD | 2 (22%) | 5 (29%) |  | 2 (22%) | 5 (29%) |  | 2 (18%) | 5 (33%) |  | 2 (18%) | 5 (42%) |
| PD | 2 (22%) | 11 (65%) |  | 2 (22%) | 11 (65%) |  | 5 (45%) | 8 (53%) |  | 3 (27%) | 5 (42%) |
| Response rate |  |  |  |  |  |  |  |  |  |  |  |
| ORR (%) ^†^ | 5 (56%) | 1 (6%) |  | 5 (56%) | 1 (6%) |  | 4 (36%) | 2 (13%) |  | 4 (36%) | 2 (17%) |
| DCR (%) ^§^ | 7 (78%) | 6 (35%) |  | 7 (78%) | 6 (35%) |  | 6 (55%) | 7 (47%) |  | 6 (55%) | 7 (58%) |

† ORR = (CR+PR)/(CR+PR+SD+PD)*100 (%)

§ DCR = (CR+PR+SD)/(CR+PR+SD+PD)*100 (%).

PD-L1, programmed cell death ligand 1; TPS, tumor proportion score; CPS, combined positive score; TIL; tumor-infiltrating lymphocytes; CR, complete response; PR, partial response; SD, stable disease; PD, progressive disease; ORR, overall response rate; DCR, diseased control rate
